# Removal of chromium from tannery industry wastewater using iron-based electrocoagulation process: experimental; kinetics; isotherm and economical studies

**DOI:** 10.1038/s41598-023-46848-9

**Published:** 2023-11-10

**Authors:** Heba A. El-Gawad, Gamal K. Hassan, Karim M. Aboelghait, Walaa H. Mahmoud, R. M. Mohamed, Ahmed A. Afify

**Affiliations:** 1 Department of Engineering Mathematics and Physics, Higher Institute of Engineering, El- Shorouk Academy, Cairo, Egypt; 2 Water Pollution Research Department, National Research Centre, 33 Behooth St, P.O. Box 12622, Dokki, Giza, Egypt; 3 Chemistry Department, Faculty of Science, Cairo University, Giza, 12613 Egypt; 4 Chemical Engineering Department, Canal Higher Institute of Engineering and Technology, Suez, Egypt

**Keywords:** Environmental sciences, Chemistry, Engineering

## Abstract

Chromium is a hazardous compound from industrial processes, known for its toxicity, mutagenicity, teratogenicity, and carcinogenicity. Chemical methods are efficient but cost-effective alternatives with reduced sludge are sought. Electro-coagulation, utilizing low-cost iron plate electrodes, was explored for factual tannery wastewater treatment in this manuscript. Operating parameters such as initial chromium concentration, voltage, electrode number, operating time, agitation speed and current density has been studied to evaluate the treatment effeciency. Under optimal conditions (15 V, 0.4 mA/cm^2^, 200 rpm, 330 ppm chromium, 8 iron electrodes with a total surface area of 0.1188 m^2^, 3 h), chromium elimination was 98.76%. Iron anode consumption, power use, and operating cost were 0.99 gm/L, 0.0143 kW-h/L, and 160 EGP/kg of chromium eliminated, respectively. Kinetics studies were pursued first-order reaction (97.99% correlation), and Langmuir isotherms exhibited strong conformity (Langmuir R^2^: 99.99%). A predictive correlation for chromium elimination (R^2^: 97.97%) was developed via statistical regression. At HARBY TANNERY factory in Egypt, industrial sewage treatment achieved a final chromium disposal rate of 98.8% under optimized conditions.

## Introduction

Wastewater treatment is no longer a mere option but an imperative necessity to safeguard the environment, ecosystems, and public health^1–3^. The tannery industry, one of the oldest worldwide, generates a significant volume of hazardous waste, laden with chemicals, salts, dyes, and other pollutants^4^. Among these pollutants, chromium stands out as a major inorganic contaminant, notably in tannery effluents with concentrations ranging from 10 to 1000 mg/L, far exceeding discharge limits^5,6^. The oxidation of chromium to its toxic form, Cr (VI), poses severe health and environmental risks^7–9^. Tannery effluents contain a cocktail of substances, including dyes, organic compounds, acids, alkalis, tannins, and various chemicals, which are not completely bound to the skins and consequently remain in the wastewater. A substantial portion of these effluents, around 90%, finds its way into the environment and the prove for this truth that the landfill leachate that formed after digestion of municipal solid waste contain a percent of chrouim^10,11^.

To address this critical issue, various methods have been employed for chromium removal from wastewater including ion exchange, chemical reduction and precipitation, reverse osmosis, photocatalytic processes, and adsorption^12–14^. The coagulation/flocculation process is one of them and has grown in popularity due to its ease of use, however its major drawback is that it employs too many chemicals, which results in secondary contamination^15,16^. Adsorption and ion exchange methods are expensive and have limited removal capacity. Thus, there is a need for cost-effective treatment methods that can handle the high chromium loads in tannery wastewater without secondary pollution. Among these methods, electro-coagulation (EC) has gained attention due to its eco-friendliness, cost-effectiveness, unlike traditional procedures, and minimal chemical usage, reducing the risk of secondary contamination^17–19^. EC involves the generation of coagulants during the degradation of sacrificial anodes due to the applied current, coupled with the production of hydrogen at the cathode, facilitating pollutant removal through precipitation and flotation. The prime drawback of this technique is the high energy consumption, which we overcme in our investigation by adding electrolyte to enhance solution conductivity and reduce energy use during electro-coagulation.

This technology has been applied successfully in treating wastewater from diverse industrial sectors, including tanneries**,** dairy wastewater**,** pharmaceutical wastewater**,** and distillery wastewater^20–23^. The efficiency of the EC process depends on several key parameters, such as electrode material, current density, wastewater pH, operating time, and initial chromium concentration. Studies have shown that optimizing these parameters can lead to significant chromium removal rates, offering potential for recycling chromium-rich sludge and using as a raw material in many industries, such as the ceramic industry and conversion of this sludge to a value-added product such as biogas energy which will also reduce any negative environmental effects^24,25^**.**

In previous studies, Nahid M. Genawi^26^ achieved complete chromium removal at 13 mA/cm^2^, pH 7, and a chromium concentration of 750 ppm, with XPS analysis showing 79.28% chromium oxides and 20.72% chromium hydroxides, indicating recycling potential^26^. Angel Villabona-Ortíz^27^ improved electro-coagulation with longer residence times, lower voltages, and increased electrode numbers, achieving a 92.9% removal rate using 10 electrodes at 20 V for 30 minutes^27^. Hamadan and El-Naas^28,29^ attained complete chromium removal at 7.9 mA/cm^2^ and enhanced the process using an EC column with a helical iron cathode and air injection for better mixing^28,29^. Other studies reports that increasing current density, enhancing electrode dissolution and removal rates, demonstrating effective chromium removal under both alkaline and acidic pH conditions^30–32^.

This manuscript focuses on the optimization of an electro-coagulation process for efficient and economical chromium elimination from simulated tannery wastewater using a simple batch electro-coagulation cell. The iron plates were utilized as electrodes for the electro-coagulation technique. The numerous operating factors like initial chromium concentration, applied voltage, current density, number of electrodes, agitation velocity, and treatment time were assessed to define the best chromium elimination. A kinetic study has been fulfilled to ensure the influence of numerous processing variables on chromium disposal. The manuscript also estimates electrode and energy consumption under ideal conditions and characterizes the deposited sludge resulting from the electro-coagulation process. After that, application of electro-coagulation on elimination of chromium from Factual tannery wastewater huddled from an effluent stream of leather tanning industry by HARBY TANNERY factory in Rubiki (Badr city) was explored under these optimized conditions. This research offers valuable insights into an environmentally friendly and efficient method for tackling chromium pollution in tannery effluents. In addition, the using of modeling isotherm with these such combinations of economical, kinetics and using real waste may decrease the effort and cost that could be paid for doing such pilot-scale work for electrochemical treatment of tannery wastewater.

## Materials and methods

### Materials

The reagents utilized in this experiment were potassium chromate of 98% purity, hydrochloric acid (HCl) of 30% purity, sodium hydroxide Pellets (NaOH) with a purity of 98% and sodium chloride (NaCl) of 98% purity. All reagents were obtained from El-Gomhouria Company for Trading Chemicals and Medical Appliances located at greater Cairo in Egypt.

### Samples collection and classification

Factual tannery wastewater samples are assembled from a leather tanning factory in the industrial area of Rubiki (east of Cairo). These samples are transported and stored at 4 ^0^C to be analyzed per APHA 2017 standards^1^. To assess disposal efficiency under varying conditions, we created a chromium stock solution by dissolving 98% pure potassium chromate salt in distilled water. We achieved the desired experimental concentrations through successive dilutions with distilled water. Table 1 reveals the factual tannery wastewater characterizations.

**Table 1:** Characterization of factual tannery wastewater.

Parameter	pH	TSS (mg/L)	COD (mg/L)	BOD (mg/L)	NH_4_ (mg/L)	Cr (mg/L)	TKN (mg/L)	H_2_S (mg/L)	Electrical conductivity (mS/cm)
Value	6–9	2700	4052	1750	100	3300	250	350	61.8

### Electro-coagulation system

**Figure 1:**
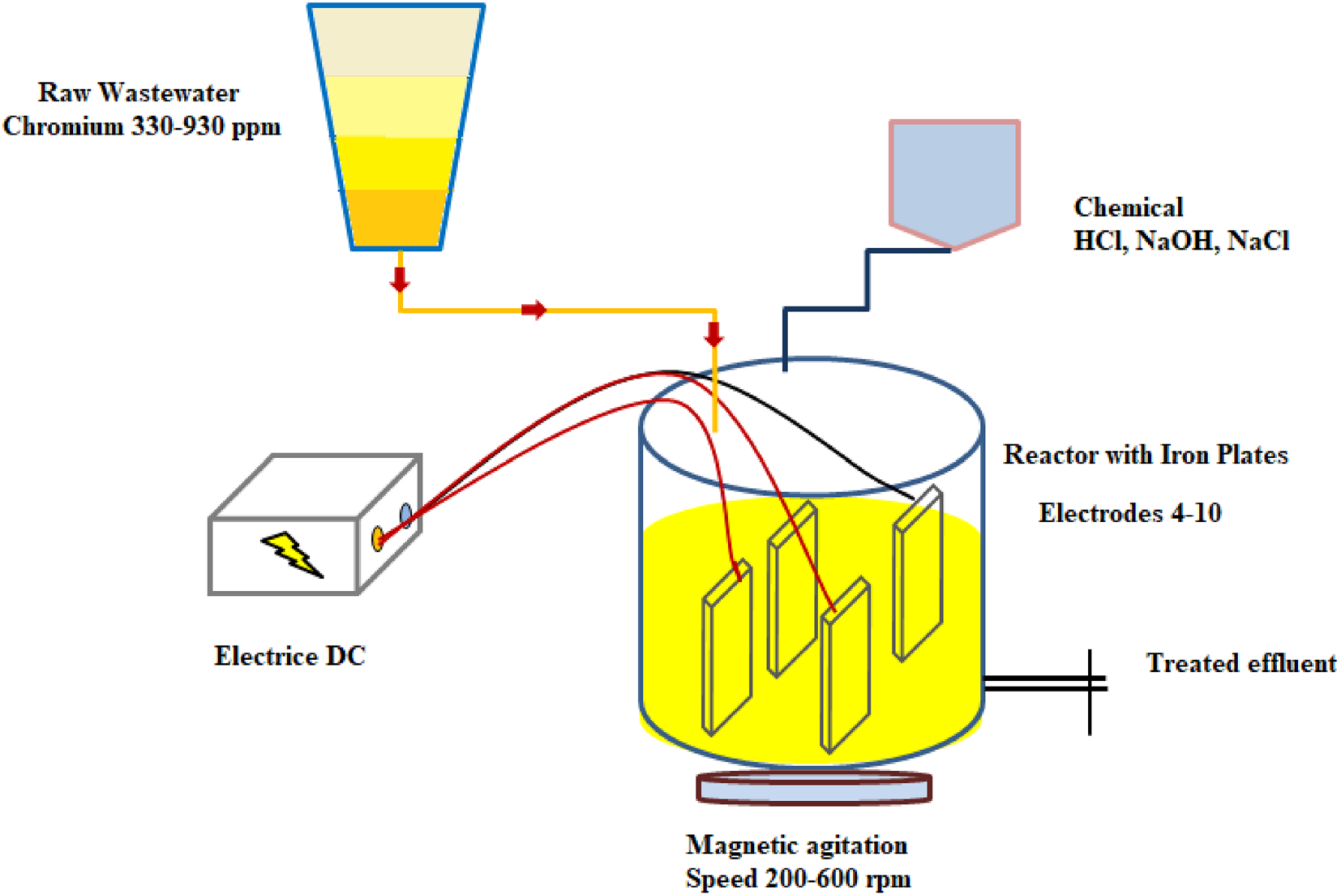
Schematic diagram of the used system for electro-coagulation.

The experimental unit as shown in Fig. 1 includes a cylindrical glass container (reactor) with internal diameter of 10.5 cm and total a capacity about 1.2 L. This design allows setting 10 equidistant iron electrodes. Each iron electrode has a rectangular Sect. (13.5 cm × 5.5 cm) with thickness of 1 mm. Electrodes are vertically positioned, and arranged parallel to each other with a space of 1cm between them and installed at 2 cm from the bottom of the reactor. The electrodes operate in monopolar mode and connect to the positive and negative depots of the DC power supply (Range 230 V/50 A). The magnetic stirrer is utilized to maintain an unchanged composition and avoid the association of the clumps in the solution.

The anode efficient area (EA) can be calculated as shown in Eq. (1)^33^**.** The immersed length here will be 9 cm form plate height (13.5 cm).

1 $${\text{EA}} = {\text{Number of}} {\text{ anod electrods}} \times 2 \left( {{\text{face}}} \right) \times {\text{immersed}} {\text{length}} \times {\text{plate}} {\text{width}}$$

### Experimental proceeding

In each trial, 500 mL of the sample was placed in the glass reactor. Optimum pH for chromium elimination is preferred within the range 4–8, as suggested by previous studies^34,35^. The observed pH increase is owin to the generation of hydroxide ions (OH^-^) from the cathode through water electrolysis, as described in Eq. (3), which is used to produce hydroxides or bind to the sludge^36^. Elevated pH values, primarily Fe(OH)_3_, positively impact contaminant removal, consistent with many litrature. OH^-^ ions can also undergo partial combination with Cr^3+^ ions, resulting in the formation of the insoluble hydroxide precipitate Cr (OH)_3_. Interestingly, when the initial pH was raised to 9.00, the efficiency of total Cr removal decreased because of the conversion of Cr ions to CrO_4_^2–^, which in turn weakened the effectiveness of Cr disposal^37^. The solution pH is adjusted by adding HCl (10%) and/or NaOH (10%) solutions as required. A specific concentration of NaCl (98% purity) is added for supporting electrolyte and depending on the experiment to increase the conductivity. All experiments were carried out at room temperature (25 ^0^C). At specified time intervals; 30 mL supernatant was withdrawn by a pipette from the top of the reactor for chromium measurement. The final concentration of chromium in the solution was determined by UV–VIS spectrophotometer; model UV-BK1900 with a 1 cm quartz cell at 540 nm. The produced sludge was analyzed by XRF analyzer. Table 2 indicates the removal efficiency combined to the change in a lot of factors to investigate the reaction optimum conditions. The chromium removal percent was appraised using next equation:

2 $$\% removal = \, [C_{0} - C_{t} ]/C_{0} *{1}00$$

where *C*_0_ and *C*_*t*_ are the initial and the final value of chromium, (mg/L).

**Table 2:** Experimental range of test factors studied.

Studied factor	Ranges
Treatment time, hour	1–5
Chromium concentration, ppm	330–930
Applied voltage, V	5–20
Current density, mA/cm^2^	0.15–0.54
Number of Fe electrode	4–10
Magnetic agitation speed, rpm	200–600

### Chromium elimination mechanism

Elimination process of chromium is done according to the oxidation and reduction reaction at anode and cathode respectively according to the next equations

3 $$2{\text{H}}_{2} {\text{O}} + 2{\text{e}} \to {\text{H}}_{2} \uparrow \left( {\text{g}} \right) + 2{\text{OH}}^{ - } \quad \left( {{\text{Cathode}}} \right)$$

4 $${\text{Fe}}\left( {\text{s}} \right) \to {\text{Fe}}^{ + 2} \left( {{\text{aq}}} \right) + 2{\text{e}}^{ - } \quad \left( {{\text{Anode}}} \right)$$

5 $${\text{CrO}}_{4}^{ - 2} + 3{\text{Fe}}^{ + 2} + 4{\text{H}}_{2} {\text{O}} \to {\text{Cr}}^{ + 3} + 3{\text{Fe}}^{ + 3} + 8{\text{OH}}^{ - } \quad \left( {{\text{sloution}}} \right)$$

6 $${\text{Cr}}^{ + 3} + 3{\text{OH}}^{ - } \to {\text{cr}}\left( {{\text{OH}}} \right)_{3} \left( {\text{s}} \right) \quad \left( {{\text{Precipitation}}} \right)$$

7 $${\text{Fe}}^{ + 3} + 2{\text{OH}}^{ - } \to {\text{Fe}}\left( {{\text{OH}}} \right)_{3} \left( {\text{s}} \right)\quad \left( {{\text{Precipitation}}} \right)$$

### Economical study

In this manuscript, plate material and electrical power price were considered as main price terms in the processing costs estimation (EGP/gm chromium eliminated) utilizing the succeeding equation electrodes consumption can be calculated from the next Eq.^38,39^**.**

8 $${\text{m}}_{{{\text{Fe}}}} = \frac{{{\text{I}} \times {\text{t}} \times {\text{M}}}}{{{\text{Z}} \times {\text{F}} \times {\text{V}}}}$$

where I = current intensity (A), t = elimination time (s), M = iron atomic weight (55.845 gm/mole), z = number of electrons transmitted in the reaction Fe = Fe^2+^  + 2e^-^, F = Faraday’s constant (96,500 Cb/mole), and V = sewer water solution volume (L).

Power exhaustion,* E* is estimated from the below equation at the optimum processing factors^40,41^.

9 $${\text{E}} = \frac{{{\text{I}} \times {\text{t}} \times {\text{U}}}}{{\text{V}}}$$

where I = current intensity (A), t = time (h), V = wastewater solution volume (L), and U = applied voltage (volt).

10 $${\text{Processing}}\;{\text{cost}} = \left( {a \times \frac{{\text{E}}}{{{\text{Chromium}}\;{\text{removed}} \left( {\text{g}} \right)}}} \right) + \left( {b \times \frac{{{\text{m}}_{{{\text{Fe}}}} }}{{{\text{Chromium}}\;{\text{removed }}\left( {\text{g}} \right)}}} \right)$$

where a is the electrical power price, (EGP/kWh) and it taken as 1 kWh = 1.60 EGP, **b** is the plate material price, (EGP/t_iron_) the price 1 t of iron = 27,500 EGP**.**

### Ethics approval

Not applicable. This manuscript does not involve researching about humans or animals.

### Consent to participate

All of the authors consented to participate in the drafting of this manuscript.

## Results and discussion

### Influence of treatment time on chromium concentration

**Figure 2:**
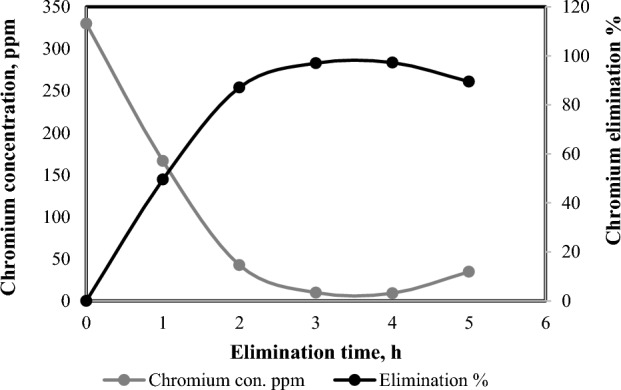
Effect of treatment time on chromium elimination efficiency (6 electrodes, 15 V, 0.4 mA/cm^2^, 200 rpm and 330 ppm Cr^+^).

The required treatment time has a significant effect not only in effluent quality but also in the treatment cost and reactor volume^42^**.** The impact of reaction time on chromium elimination efficacy was inspected under several process time values ranging from 1 to 5 h, while other operating factors were kept at constant values; i.e. 15 V, 0.4 mA/cm^2^ current density, 330 ppm initial chromium concentration, 200 rpm, and 6 Fe electrodes. It is obvious as shown in Fig. 2 that the elimination efficacy rises with the increase in treatment time. 3 h were enough to achieve removal efficiency of 97.01% which also almost equal to maximum the removal efficiency after 4 h 97.25%. Economically, time reaction 3 hours should be considered. The elimination efficacy reduces with increasing time which may be because of the influence of the electro-coagulation attaining the saturation point. Thus, unrestricted progress is not accomplished by raising the reaction time. As time increases, metal sheets develop a thin protective passivation film, affecting the quantity of deteriorated Fe(II) plates, generating radicals, reducing ions, and decreasing flocculant levels, while also diminishing the oxidation effect. Another crucial factor to consider in electro-coagulation is power consumption, influenced by longer operation times, leading to higher treatment costs. This is mainly due to the rapid oxidation of Cr^3+^ and the formation of Cr(OH)_3_. Initially, fine particles tend to aggregate during electro-coagulation, but as treatment time extends, particle size increases, resulting in reduced chromium disposal efficiency^43^**.** One possible explanation is that after 8 h, deposited chromium returns to the liquid phase, leading to decreased chromium disposal efficiency and an increased ratio of total dissolved solids.

### Influence of initial chromium concentration on treatment efficiency

**Figure 3:**
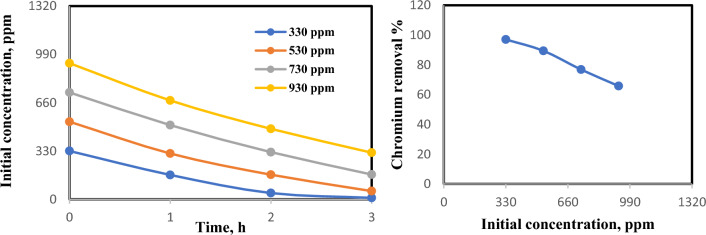
Effect of initial chromium concentration on chromium elimination efficiency (6 electrodes, 15 V, 0.4 mA/cm^2^, 200 rpm and 3 h).

Figure 3 indicates that the disposal efficiency reduces with a raise in an initial chromium concentration. This performance is a result of the lack of clumps for a sorption of extra chromium at elevated concentrations and likewise because of the minimum iron corrosion rates and rising iron surface passivation at elevated chromate concentrations^44,45^. The cause why the chromium elimination efficacy reduces with raising in its premier concentration is concluded from Faraday’s law. In line with Faraday's law, a fixed current density yields a steady release of Fe^2+^ into the solution through anodic electro-dissolution, which intensifies with prolonged electrolysis^46,47^. These ions play a a pivotal role in facilitating the reduction of Cr(VI) to Cr(III), leading to the formation of insoluble Cr(OH)_3_ and Fe(OH)_3_. Subsequently, when the initial chromium concentration rises, a substantial quantity of iron ions becomes essential during prolonged electrolysis periods to achieve complete chromium ion reduction^29,48^. Alternatively, one can increase either the current density or the electrode surface area proportionally to ensure sufficient Fe^2+^ production for effective Cr(VI) disposal from the effluent^49^.

### Influence of applied voltage and current density on treatment efficiency

**Figure 4:**
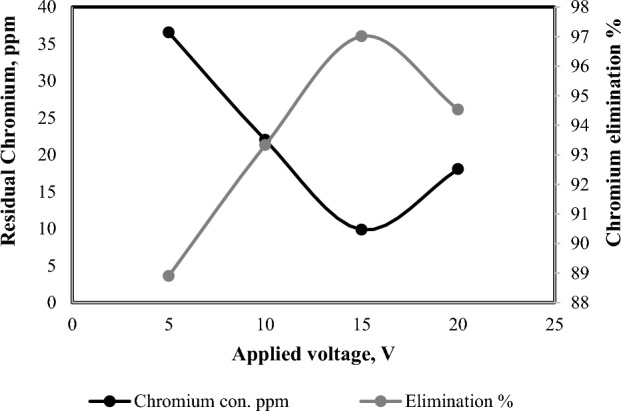
Effect of applied voltage on chromium elimination percentage (6 electrodes, 0.4 mA/cm^2^, 330 rpm, 330 ppm Cr^+^ and 3 h).

The applied voltage is a crucial parameter that plays a critical role in the electro-coagulation technique. The data depicted in Fig. 4 exhibits that an increase in applied voltage corresponds to a greater degree of chromium disposal.The maximum elimination percentage was 97.01% at voltage of 15 V then there was a decrease in the elimination percentage with additional voltage. For that reason, the applied voltage was elected 15 V for chromium elimination during the electro-coagulation technique. Hasan et al.^42^ reported the similar observation and achieved utmost chromium efficacy at 15 V.

**Figure 5:**
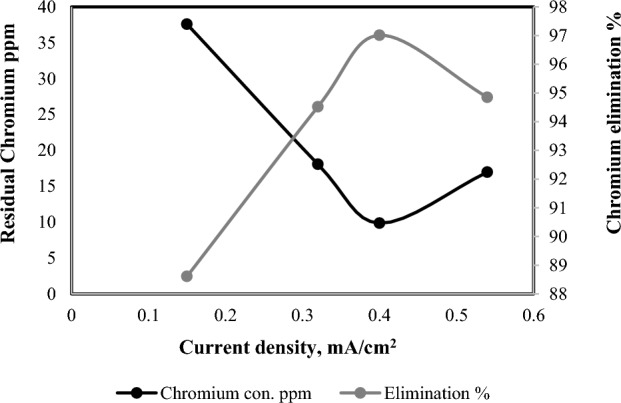
Effect of current density on chromium elimination percentage (6 electrodes, 15 V, 200 rpm, 330 ppm Cr^+^ and 3 h).

Increasing current density enhances metal removal efficiency by boosting Fe^2+^ and OH^-^ production^40,50^. Current density impacts electro-chemical metal dosing rate, electrolytic bubble production, and floc growth,while also influencing electrical energy consumption, electrode material usage, and overall operating costs in electro-coagulation^51,52^. The same results were in the current density as shown in Fig. 5. The chromium elimination percentage is raised with raising the current density. In accordance with Faraday’s law, this observation was respected owing to an anodic dissolution^28,39,45^. In contrast, when a current density of 0.54 mA/cm^2^ is applied, a slight reduction in the elimination percentage is noticed. This reduction can be attributed to the formation of a higher amount of hydrogen bubbles at the cathode, which causes the sludge to rise and hinders the formation of flocs^28,53,54^. As a result, the chromium removal percent is reduced. It is noticed that with raising the current density, the turbidity of wastewater was also raised.

As per Moussa et al. 's findings in (2017)^55^, this behavior can be ascribed to a critical current density threshold. Even when using higher current density, the treated water does not exhibit a significant improvement beyond this critical point. Elevated current densities result in the release of a notable quantity of Fe^3+^ ions through anode dissolution. As a result, the generated Fe(OH)_3_ molecules adhere to surfaces without the presence of contaminants due to the saturation of adsorption sites created by iron hydroxide^56^. The utilization of high working current values has been linked to the generation of residual energy, leading to an increase in water temperature^7^. For that reason, 0.4 mA/cm^2^ of current density believed the suitable current density for the technique electro-coagulation.

**Figure 6:**
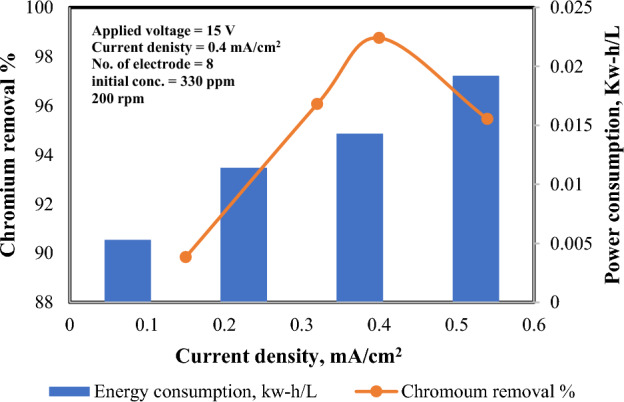
Effect of current density on power consumption and chromium removal % (8 electrodes, 15 V, 200 rpm, 330 ppm Cr^+^ and 3 h).

The impact of current desity on the power consumption was also elucidated under several values ranging from 0.15 to 0.54 mA/cm^2^ while other operating factors were kept at constant values; i.e. 15 V, 330 ppm initial chromium concentration, 200 rpm, and 8 Fe electrodes for 3 h. It is obvious as depicted in Fig. 6 that the power consumption is directly related to the current density. Increasing current density from 0.15 to 0.4 mA/cm^2^ boosted chromium disposal efficacy from 89.84% to 98.76%, with power consumption rising from 0.0053 to 0.0143 kW-h/L. In a separate study, raising current density from 0.42 to 0.94 mA/cm^2^ improved chromium removal efficiency from 74.35% to 100%, alongside an energy consumption increase from 0.24 to 0.94 kW-h/m^3^.

### Effect of magnetic rotational speed and number of fe electrodes on treatment efficiency

**Figure 7:**
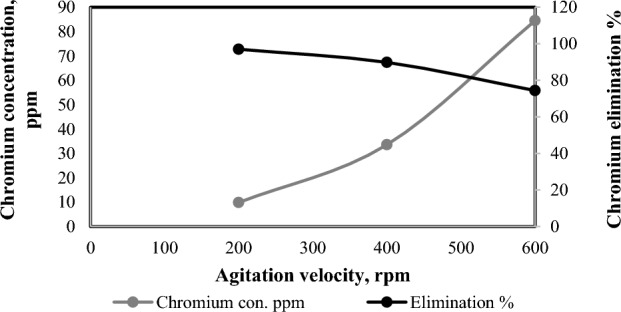
Effect of magnetic agitation velocity on chromium elimination percentage (6 electrodes, 15 V, 0.4 mA/cm^2^, 330 ppm Cr^+^ and 3 h).

Rotational speed is very important as it ensures that the flocculants from the dissolved electrodes are homogeneously dispersed in the reactor. Referring to Fig. 7, the suitable magnet agitation velocity can be 200 rpm. In the chromium elimination process, iron and hydroxyl ions combine to create highly absorbent iron hydroxide that binds to contaminants. Iron hydroxide also forms aggregates with a network structure, effectively removing contaminants from the liquid. Excessive agitation can disrupt these aggregates, releasing contaminants^57^. Also, high agitation speeds lead to increased shear rates at the floc interface, resulting in irreversible floc breakage and preventing re-growth^58^.

**Figure 8:**
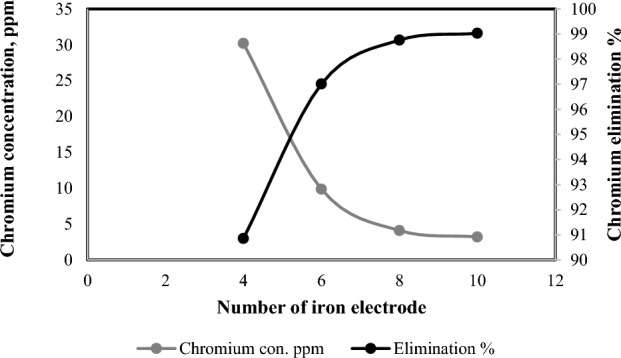
Effect of number Fe electrodes on chromium elimination percentage at (0.4 mA/cm^2^,15 V, 200 rpm, 330 ppm Cr^+^ and 3 h).

The effects of rising electrode number on the elimination efficacy are elucidated in Fig. 8. From the figure, it was noticed that the disposal efficiency was raised with raising number of Fe electrodes from 4 to 8 electrodes. This elimination is owing to the destabilization of Cr in the solution through the hydrolysis products of iron, which permits accumulation and a superior segregation of the solution thru sedimentation or flotation.

It was noticed that the simultaneous elevate in the plates number improves disposal efficacy. This could be owing to the existence of ferrous hydroxides, which raises with the plates number. These composites perform as coagulants and trap contaminant molecules, perhaps thru the relative rise in Fe(II) hydroxides beside the plates number^59^. The outcomes also indicate that elevating the effective area enhanced the disposal efficiency and reduced processing time beloved, the elucidation of this observation as obeys: greater plate surface area led to a greater dispersion of bubbles everywhere the reactor, whilst a minimal plate surface area led to a massive bubbles source inside the reactor, and as the bubbles dispersion inside the reactor, the likelihood of collision between the bubbles and coagulant rises causing to rise the elimination efficacy.

The outcomes also indicated that the elimination efficiency relatively constant in case of 10 electrodes applied. For that reason, the ideal number of electrodes was considered 8 electrodes for chromium elimination through the electro-coagulation process.

### Kinetic investigation via treatment process

The kinetic models of Cr elimination from wastewater were fulfilled under the ideal processing factors (330 ppm premier chromium concentration, 0.4 mA/cm^2^ current density, elimination time of 3 h, 8 iron electrodes, 200 rpm, and applied voltage of 15 V). Adequate results of Cr elimination kinetics are revealed in Table 3. The linear formula of first and second order kinetic models can be introduced in the following equations:

11 $${\text{Ln}}\;{\text{C}}_{t} = {\text{K}}_{1} {\text{t}}$$

12 $$\frac{1}{Ct} - \frac{1}{Co} = {\text{K}}_{2} t$$

**Table 3:** Reaction rate of 330 ppm initial chromium concentration.

Reaction rate equation	Kinetic equation	Rate constant	R^2^
First order equation	ln C_t_ = 0.0253 t	0.0253 min^−1^	0.9799
Second order equation	$\frac{1}{Ct} - \frac{1}{Co} =$ 0.001 t	0.001 L min^−1^gm^−1^	0.8067

where C_o_ and C_t_ are the premier and final chromium concentrations, respectively. K_1_ and K_2_are first and second order rate constants in min^−1^ and L. gm^−1^ min^−1^, respectively, and t is the treatment time (in min). A plot lnC_t_ and [$\frac{1}{Ct} - \frac{1}{Co}$] against time for each run leads to a straight line whose slope is K_1_ and K_2_, respectively. It can be observed that the correlation coefficient R^2^ of the pseudo first order kinetic model was better than that of the pseudo second order kinetic model. An identical examination has been mentioned before by^37^ in the elimination of chromium from tannery wastewater by electro-coagulation. Also, Bingül et al.^60^ and Lorgio Valdiviezo-Gonzales et al.^7^ reported the first order kinetic model with good correlation for chromium. The regression analysis of the concentration curves against treatment time denotes that the reaction rate can be depicted by means of first order kinetic model for chromium elimination. The end result is exhibited in Fig. 9.

**Figure 9:**
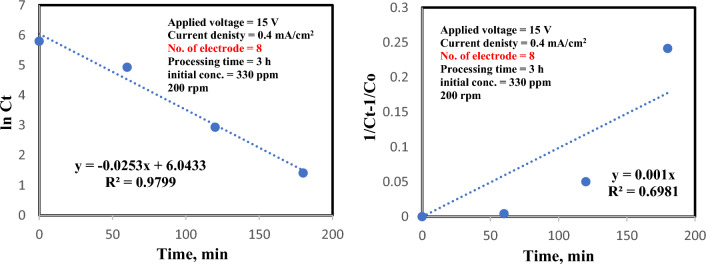
Reaction rate of 330 ppm initial chromium concentration at ideal processing factors. **a** First order kinetic model. **b** second order kinetic model.

### Isothermal modeling investigation

Isotherms are employed to depict the adsorption process under ideal factors ((330 ppm premier chromium concentration, 0.4 mA/cm^2^ current density, 8 iron electrodes, 200 rpm, and applied voltage of 15 V), and the Langmuir and Freundlich isotherms are widely used for this purpose. The Langmuir isotherm specifically characterizes adsorption at uniform sites, where a monolayer is formed. It is represented by a linear equation, given by the below Eq. (13)^49^:

13 $$\frac{{{\text{Ce}}}}{{{\text{qe}}}} = \frac{1}{{{\text{qm}} \cdot {\text{KL}}}} + \frac{{{\text{Ce}}}}{{{\text{qm}}}}$$

**Figure 10:**
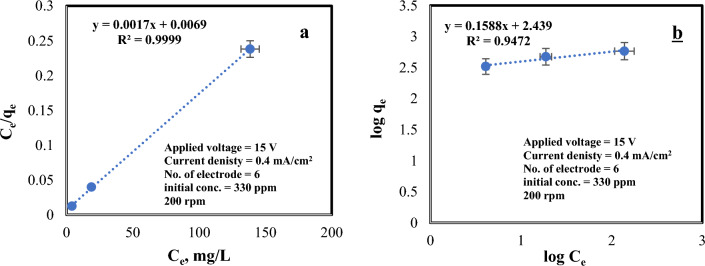
Isothermal models for the chromium adsorption onto carbonized rice straw: **a** Langmuir isotherm model. **b** Freundlich isotherm model.

The adsorption capacity q_e_ (mg/gm) was calculated by means of the bellow Eq. (14):

14 $${\text{q}}_{{\text{e}}} = \left[ {\left( {{\text{C}}_{0} {-}{\text{C}}_{{\text{e}}} } \right) \cdot {\text{ V}}} \right]/{\text{w}}$$

where q_e_ (mg/gm) is the equilibrium concentration of chromium, C_e_ (mg/L) is the equilibrium concentration in the liquid phase, V and W are volume of solution (L) and the weight of a sorbent (gm), individually. A linear plot of C_e_/q_e_ versus C_e_ indicates that the adsorption data reasonably fits the Langmuir isotherm, as exhibited in Fig. 10. The constants were obtained from the slope (1/q_m_) and intercept (1/K_L_) and are listed in Table 4. The Langmuir equation, expressed in terms of the dimensionless factor R_L_, is expressed by:

15 $${\text{R}}_{{\text{L}}} = 1/\left( {1 + {\text{b}}_{{\text{L}}} \cdot {\text{C}}_{0} } \right)$$

**Table 4:** Results of isotherms for chromium adsorption of onto carbonized rice straw.

Langmuir isotherm model					Freundlich isotherm model		
q_m_, mg/gm	K_L_, gm/L	1/b_L_, L/mg	R_L_	R^2^	1/n	K_f_, mg/gm	R^2^
588.235	0.2464	0.0069	2.091*10^–5^	99.99%	0.1588	274.79	94.72%

The value of R_L_, a positive number (0 < R_L_ < 1), denotes the feasibility of the sorption process.

The Freundlich isotherm model is based on the assumption that adsorption takes place on heterogeneous surfaces with varying adsorption energies. The linear form of the Freundlich equation is expressed as:

16 $${\text{Log}}\;{\text{q}}_{{\text{e}}} = \log \,{\text{K}}_{{\text{f}}} + 1/{\text{n}}\;\log \;{\text{C}}_{{\text{e}}}$$

where 1/n is the heterogeneity factor related to intensity, and K_f_ is the Freundlich constant. The slope and intercept of log q_e_ against log C_e_ give 1/n and K_f_ values as elucidated in Fig. 10b.

The comparison of correlation values (R^2^) between the Langmuir and Freundlich adsorption isotherms indicates that the Langmuir isotherm outperforms the Freundlich isotherm. This indicates a robust correlation between the experimental data and the Langmuir model, suggesting that the Langmuir adsorption isotherm accurately describes the adsorption behavior in the studied system. The Langmuir isotherm is consistent with prior work^61^.

### Sludge characteristics

The formed sludge was gathered, dried at 103 ^0^C for 24 h, and then cooled in desiccators to assess the surface morphology of the deposited sludge throughout the XRF analysis. The XRF results showed that the chemical composition of the sludge ranged from 70–75% as weight of iron oxide and the percent of the chromium was ranged from 25–30% as weight from the simulated wastewater and the ferric and ferrous hydroxides formed during electro-coagulation later turn into magnetite. These results were in the same line of Un et al.^30^who stated that the percent of the chromium in the produced sludge in his study was 16.6% as weight and 74.3% as weight of chromium which confirms our finding and our hypothesis of iron oxide forming.

### Statistical analysis for the proposed treatment process

To elucidate the influence of the operative factors on the chromium elimination efficiency, a mathematical correlation must be recommended. Statistical and least square multivariate regression techniques are extremely utilized for modeling and analysis of troubles in which a response of interested dependent variable is influenced by numerous independent variables. This model was utilized in an attempt to detect the mathematical correlation that can clarify the influence of the operative factors on the chromium disposal efficiency (ANOVA).

The values and p-values of the coefficients are provided in Table 5. The p-values decides if any given factor is momentous or not. The correlation terms having a p-value less than 0.0001 are momentous. The R^2^ was 97.97%; it exhibited that the changeability in the adsorption could be explicated by the model, with the cohesion between the experimental and predicted values being momentous inside the process. The acquired correlation in terms of momentous factors only has the following formula:

17 $${\text{Removal}}\,\% = 167.24 + 13.68\,{\text{CD}}{-}0.0687{\text{C}}_{0} {-} \, 0.04987\,{\text{AV}}{-}23.14\;{\text{N}} + 3.941\;{\text{t}}{-}48.3636\;{\text{CD}}^{2} { + }8.008\;t*N{-}6.506\;{\text{t}}^{2}$$

where CD is the current density, C_o_ is the chromium initial concentration, AV agitation velocity, t is the elimination time, and N is the number of iron electrodes.

**Table 5:** Value and *p* value of all coefficients.

	Coefficients	*p *Value	Significance
Intercept	167.2390034	2.1427E−05	
X Variable 1	0.524511859	0.09103226	Not significant
X Variable 2	13.68217639	0.000014517	Significant
X Variable 3	− 0.068652401	0.00011569	Significant
X Variable 4	− 0.049873034	0.00016299	Significant
X Variable 5	− 23.1404072	2.6584E−06	Significant
X Variable 6	3.940721154	9.69936E−10	Significant
X Variable 7	− 48.36362085	2.37E−06	Significant
X Variable 8	− 2.92521E-05	0.435155013	Not significant
X Variable 9	0.135376449	0.398504319	Not significant
X Variable 10	8.008042517	8.62371E−07	Significant
X Variable 11	− 6.50637585	3.73777E−06	Significant

**Figure 11:**
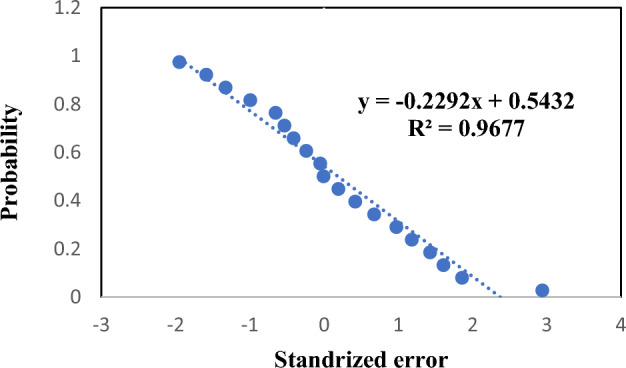
Normal probability of standardized errors.

**Figure 12:**
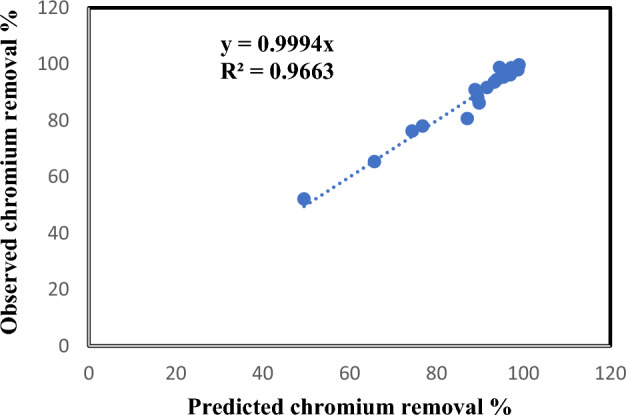
Predicted chromium removal % vs. observed chromium removal %

The normal probability of standardized residuals with average correlation errors of zero is demonstrated in Fig. 11. The linear distribution of the residual errors elucidates that the errors are normally distributed which denotes that the model prognoses are not prejudiced and for more clarity the comparison between the experimentally observed values of chromium removal percentage and predicted values is shown in Fig. 12. The figure exhibits a perfect convention between them. Table 6 clarifies the experimental observed and predicted chromium elimination percentage for the 19 runs. The chromium elimination percentage varied from 49.53% to 98.79%.

**Table 6:** Experimental observed and predicted chromium elimination percentage.

Runs	Observed %	Predicted %
1	88.9	90.9225593
2	93.33	93.54511859
3	97.01	96.16767789
4	94.53	98.79023719
5	91.64	91.64911875
6	95.43	95.37948421
7	96.07	96.05741731
8	89.4	88.23594914
9	76.8	77.96405086
10	65.74	65.35198305
11	89.83	86.19307116
12	74.4	76.21846442
13	93.88	94.40023719
14	98.76	97.93511859
15	99.03	99.7025593
16	49.53	52.12217449
17	87.09	80.65130204
18	97.25	98.67130204
19	89.5	88.16217449

### Application of electro-coagulation on chromium disposal from factual tannery wastewater with economical analysi

Chromium removal from sewage obtained from HARBY TANNERY in Rubiki (Badr city) was studied. The initial characteristics of the tannery sewage were 2700 mg/L total suspended solids, 1750 mg/L BOD5, 4025 mg/L COD, pH (6–9), NH4 (100 mg/L), TKN (250 mg/L), oil and gas (140 mg/L), sulphide (350 mg/L), and 3300 mg/L chromium. Electro-coagulation was conducted under optimized conditions (15 V applied voltage, 0.4 mA/cm^2^ current density, 200 rpm, and 330 ppm chromium initial concentration using 8 electrodes for 3 h). The real tannery wastewater concentration was reduced from 3300 to 330 ppm through dilution with distilled water. These results indicate a remarkable chromium elimination rate of 98.8% under these optimized conditions. A comparison with previous electro-coagulation studies on tannery wastewater treatment is exhibited in Table 7.

**Table 7:** Comparison of current outcome with other consequences.

Sample type	Electrode utilized	Premier chromium concentration, mg/L	Current density	Applied voltage, V	Number of electrodes	pH	Elimination time	Mixing speed, rpm	The power exhaustion	Electrode exhaustion	Processing cost	Elimination %	Refs.
Simulated wastewater	Fe	330	0.4 mA/cm^2^	15	8	4–8	3 h	200	0.0143 kWh/L	0.99 gm/L	160 EGP/kg Cr eliminated	98.76	Current consequence
Tannery wastewater	Zn/Cu	340	0.36 mA/cm^2^	15	2	–	18 h	–	–	–	–	98	Hasan et.al^42^
Aqueous solution	Fe	250	15 mA/cm^2^	–	6	–	20 min	–	18.5 kWh/m^3^	4 mg Fe/L	92.5 S.P/m^3^	98.7	^53^
Aqueous solution	Al-SS	50	–	20	10	–	30 min	–	–	–	–	92.9	Villabona-Ortíz et al. ^35^
Aqueous solution	Fe	800	40 mA/cm^2^	–	2	4–8	40 min	–	0.046 kWh/L	4.34 g Fe/L	–	99	Dermentzis et al.^40^
Aqueous solution	Fe	1000	9.34 mA/cm^2^	–	2	3.54	1 h	–	–	–	1300 TUG/m^3^	99.89	Ulambayar et al. ^65^
Electroplating effluent	Fe	Cr = 55.3, Pb = 3.5	73.5 A/m^2^	–	2	3.5	90 min	–	17.14 kWh/m^3^	–	1.86 US$/m^3^	Cr = 91.7, Pb = 91.3	Sharma et al**.** ^66^
Aqueous solution	(Al + Ti)–C	Cr^+3^ = 50, Cr^+6^ = 50	600 A/cm^2^	5		6	30 min	–	0.103 W h/g	–	–	Cr^+3^ = 98, Cr^+6^ = 91	Li et al. ^37^
Metal complex dye effluent	Fe	82.7	89.45 A/m^2^	–	2	5.83	50 min	–	2.499 kWh/m^3^	–	0.207 US$/m^3^	99.64	Taheri et al.^38^
Aqueous solution	Al	200	–	9.14	2	4.23	30 min	–	3.536 kWh/m^3^	–	–	91	Zaroual et al. ^31^

Economic studies have been used for the purpose of commercialization and many studies used the economical in their research to make the research easy use^62–64^. Based on the Eqs. (8–10), it was found that under optimized conditions (330 ppm initial chromium concentration, 0.4 mA/cm^2^ of current density, treatment time of 3 h, 8 iron electrodes, 200 rpm, and applied voltage of 15 V), the sacrificial iron anode exhaustion per liter of the effluent was 0.99 gm Fe per L. and the power exhaustion, E was 0.0143 kW-h/L of treated wastewater. The final Processing cost was (0.1546 EGP/gm (cr) removed).

A kilogram of pure chromium amounts to the equivalent of 4,500 EGP, and thus it is a profitable value for the idea of working the cell to remove and recover chromium in the future.

## Conclusion

Electro-coagulation technique was used to remove chromium from wastewater of tanning and other leather processing. The recent study aimed to determine the optimum operation conditions which achieve high removal efficiency taken into account the economic concept and it concluded that:

- For raw wastewater with 330 ppm of chromium, elimination efficacy of 98.76% can be achieved at the conditions of 15 V, 0.4 mA/cm^2^ current density, 200 rpm, 8 plate of electrodes and reaction time of 3 h.
- The chromium elimination followed the 1^st^ order reaction by kinetic analysis of electro-coagulation technique with a 97.99% correlation factor (R^2^).
- The chemical composition of the deposited sludge after treatment ranged from 70–75% as weight of iron oxide and the percent of the chromium was ranged from 25–30% as weight from the simulated wastewater and the ferric and ferrous hydroxides formed during electro-coagulation later turn into magnetite.
- The acquired correlation, considering significant factors only, can be expressed by the following formula:
  $${\text{Removal }}\% \, = \, 167.24 \, + \, 13.68\;{\text{CD }}{-} \, 0.0687\;{\text{C}}_{0} {-} \, 0.04987\;{\text{AV}}{-}23.14\;{\text{N}} + 3.941 \, t{-}48.3636\;{\text{CD}}^{2} + 8.008\;t*N{-}6.506\;{\text{t}}^{2}$$
- The maximum elimination percentage of chromium from sewage assembled from the effluent stream of the HARBY TANNERY factory in Rubiki (Badr city) reached 98.8% under the aforementioned optimal processing conditions. The 98.8% removal achievable exceeds most prior electro-coagulation studies, highlighting the optimization of operating conditions.
- At optimized conditions, each 326 removed ppm from chromium consumes 0.99 gm/L from iron anode electrode, 0.0143 kw-h/L power and costs about 0.05 EGP.
- In comparison to chemical coagulation, electro-chemical treatment is a faster and more efficient and cost-effective technique for wastewater treatment, providing a sustainable treatment alternative. It requires lower coagulant doses and shorter treatment times, making it an efficient and economical choice. In addition, the using of modeling isotherm with these combinations of economical, kinetics and using real waste may decrease the effort and cost that could be paid for doing such pilot-scale work for electrochemical treatment of tannery wastewater.

## Data Availability

The datasets used and/or analyzed during the current study are available from the corresponding author upon reasonable request.
